# Infectious Diseases in Man

**Published:** 1897-10

**Authors:** 


INFECTIOUS DISEASES IN MAN.—From the Austrian Sanitary Report; period from June 21 to July 18, 1896.—Hyssa: The following were injured by the bite of mad or suspected dogs: In Istria, 1; in Bohemia, 15; in Mähren, 5; and in Bukownia, 2 persons. Of these, 5 were subjected to the anti-rabies treatment in Vienna and 2 in Bucharest. In Lower Austria 1 child died of typical hyssa which had been bitten by a dog suspected of rabies.

Anthrax: In Vienna one fatal case occurred, the details of which have not been made public. All the cases of this disease in Bohemia and Mähren had a favorable outcome. During the period from July 19 until August 15, 1896, two men who had assisted in the burial of a cow affected with splenitis were affected with anthrax-pustules. A further infection in a case of a woman in Czernowitz has been announced. The details are not yet published.

